# Racial/Ethnic Differences in Trajectories of Dementia Onset

**DOI:** 10.1093/geroni/igab046.2768

**Published:** 2021-12-17

**Authors:** Sunshine Rote, Heehyul Moon

**Affiliations:** 1 University of Louisville, Louisville, Kentucky, United States; 2 University of Louisville, university of Louisville, Kentucky, United States

## Abstract

Racial and ethnic minority older adults—especially non-Latino Black and Latino older adults—continue to have a higher prevalence of dementia with longer delays in formal diagnosis compared to non-Latino Whites. Few studies have estimated racial/ethnic differences in trajectories of dementia onset using nationally representative data with representation from the three largest racial/ethnic groups in the U.S.: non-Latino White, non-Latino Black, and Latino older adults. Additionally, given the delays in formal diagnosis we rely on a measure of probable dementia that takes into account both formal diagnosis and cognitive function. Data from the National Health and Aging Trend Study (NHATS, 2011–2019) reveals three trajectories of dementia onset (early, late, and dementia-free) and we find that Latino and Black older adults are at greater risk for early dementia onset compared to non-Latino Whites. Our next step is to explore the role of social function for dementia disparities.

